# Association of adiposity indices with cardiometabolic multimorbidity among 101,973 chinese adults: a cross-sectional study

**DOI:** 10.1186/s12872-023-03543-x

**Published:** 2023-10-21

**Authors:** Xiaoru Qin, Chaolei Chen, Jiabin Wang, Anping Cai, Xiaoxuan Feng, Xiaofei Jiang, Yingqing Feng

**Affiliations:** 1 Department of Cardiology, Zhuhai hospital affiliated with Jinan University (Zhuhai People’s Hospital), Zhuhai, China; 2 Department of cardiology, Guangdong Cardiovascular Institute, Guangdong Provincial People’s Hospital, Guangdong Academy of Medical Sciences, Southern Medical University, Guangzhou, China; 3 Global Health Research Center, Guangdong Provincial People’s Hospital (Guangdong Academy of Medical Sciences), Southern Medical University, Guangzhou, China; 4 Guangdong Provincial People’s Hospital (Guangdong Academy of Medical Sciences), Southern Medical University, Guangzhou, China; 5 Department of Cardiology, Zhuhai People’s Hospital (Zhuhai hospital affiliated with Jinan University), Zhuhai, China

**Keywords:** Adiposity indices, Obesity, Cardiometabolic multimorbidity

## Abstract

**Background:**

Cardiometabolic multimorbidity (CMM) and obesity represent two major health problems. The relationship between adiposity indices and CMM, however, remains understudied. This study aimed to investigate the associations of body mass index (BMI), waist circumference (WC), waist-to-height ratio (WHtR), a body shape index (ABSI), body roundness index (BRI), and conicity index (CI) with CMM among Chinese adults.

**Methods:**

Data of 101,973 participants were collected from a population-based screening project in Southern China. CMM was defined as having two or more of the following diseases: coronary heart disease, stroke, hypertension, and diabetes. The relationship between the six adiposity indices and CMM was investigated by multivariate logistic regression and restricted cubic splines. Receiver operator characteristic curve, C-statistic and net reclassification index were used to estimate the discriminative and incremental values of adiposity indices on CMM.

**Results:**

Logistic regression models showed the six adiposity indices were all significantly associated with the odds of CMM with non-linear relationships. For per SD increment, WC (Odds ratio [OR]: 1.66; 95% confidence interval (CI): 1.62–1.70) and WHtR (OR, 1.61; 95% CI, 1.58–1.65) were more significantly associated with a higher prevalence of CMM than BMI (OR, 1.55; 95% CI, 1.52–1.58) (all *P* < 0.05). In addition, WC, WHtR, and BRI displayed significantly better performance in detecting CMM compared with BMI (all *P* < 0.05). Their respective area under the curve (AUC) values were 0.675 (95% CI: 0.670–0.680), 0.679 (95% CI: 0.675–0.684), and 0.679 (95% CI: 0.675–0.684), while BMI yielded an AUC of 0.637 (95% CI: 0.632–0.643). These findings hold true across all subgroups based on sex and age. When Adding WC, WHtR, or BRI to a base model, they all provided larger incremental values for the discrimination of CMM compared with BMI (all *P* < 0.05).

**Conclusions:**

Adiposity indices were closely associated with the odds of CMM, with WC and WHtR demonstrating stronger associations than BMI. WC, WHtR, and BRI were superior to BMI in discriminative ability for CMM. Avoidance of obesity (especially abdominal obesity) may be the preferred primary prevention strategy for CMM while controlling for other major CMM risk factors.

**Supplementary Information:**

The online version contains supplementary material available at 10.1186/s12872-023-03543-x.

## Background

Multimorbidity, which refers to the simultaneous presence of two or more chronic illnesses in an individual, is becoming an increasingly pressing healthcare issue worldwide and a major focus of global health research [1]. Multimorbidity imposes significant burdens on both individuals and society, as demonstrated by higher rates of disability and mortality, greater incidence of psychological disorders, and increased medical expenses [2–5]. Cardiometabolic multimorbidity (CMM) is the most common and serious type of multimorbidity, characterized by the co-occurrence of two or more cardiometabolic diseases, such as hypertension, diabetes, coronary heart disease (CHD), and stroke [6]. Concerningly, the incidence of CMM is on the rise at an alarming speed [7, 8]. Considerable evidences have shown that CMM associates with higher risk of dementia, damaged quality of life, and mortality [2, 9]. Furthermore, the burden of cardiometabolic disease is rapidly increasing in low- and middle-income countries, further exacerbated by weak health and social protection systems [10]. However, preventive measures of CMM remain understudied as a major and persistent problem [7, 8], so early identification of its risk factors is crucial.

Obesity is widely acknowledged as a critical risk factor that can be modified for the prevention and management of cardiometabolic diseases [11]. For instance, a substantial quantity of data has demonstrated that the body mass index (BMI), a widely used indicator of general obesity, can predict the risk of both a single cardiometabolic disease and CMM [11–13]. However, given the limitations of BMI in accurately reflecting abdominal and visceral adiposity, it is crucial to incorporate other measures for assessing obesity [14, 15]. This is further supported by findings from the Chinese Hypertension Survey, which reported that approximately 277.8 million individuals experienced abdominal obesity from 2012 to 2015. Waist circumference (WC) and waist-to-height ratio (WHtR) are significant anthropometric markers of abdominal obesity, and they are more accurate than BMI in predicting a single cardiometabolic disease [16, 17]. To our knowledge, only one study has explored the prospective association between WC, WHtR and CMM [18]. However, the results were inconclusive as a consequence of the limited sample size. Recently, some adiposity indicators that comprehensively estimating body fat distributions such as conicity index (CI), a body shape index (ABSI) and body roundness index (BRI) have been largely used in epidemiological research and shown superior value for identifying metabolic and cardiovascular risk compared with BMI [19–22]. The relationship between single cardiometabolic disease and CMM and certain obesity anthropometric indices has been investigated in some countries and regions [18, 21, 23, 24]. However, some findings have been inconsistent, and it is still unclear which adiposity indicator is more suitable for predicting CMM. Furthermore, the relationship between obesity indices - particularly ABSI, BRI, and CI - and CMM remains understudied in low- and middle-income countries, such as China. Additionally, no research has compared the discriminatory power of obesity-related indicators for CMM in a single large Chinese population. To address this gap, our study sought to evaluate and compare the associations between multiple obesity indices and CMM within a large cross-section of the Chinese population. Our aim was also to identify optimal thresholds for these adiposity indices.

## Methods

### Study design and population

The China Patient-Centered Evaluative Assessment of Cardiac Events Million Persons Project (China-PEACE MPP) was a large screening program funded by the government, aiming to identify high-risk populations for Cardiovascular diseases across the country [25]. The design and methods of the project have already been outlined in prior literature [25, 26]. Data on participants in Guangdong province in southern China, which is a crucial component of the China-PEACE MPP, were analyzed in the present study. Following the national project [27], we used a convenience sampling strategy to select 8 sites (4 rural counties and 4 urban districts) from Guangdong Province. At each site, 5 towns or subdistricts were selected based on their size and population stability and participants were invited to the project by local staff via extensive publicity campaigns on television and in newspapers. The study included eligible individuals aged 35–75 years who had been residents of the region for at least six months preceding the screening. Overall, the study enrolled 102,358 subjects. Subjects with missing height (n = 329), weight (n = 224) and/or WC (n = 120) measurements were excluded. In the end, 101,973 individuals who have complete information including exposure variables, outcome variables, and all covariates were included in the study’s final analysis. The Guangdong Provincial People’s Hospital Ethics Committee granted approval for this study (No. GDREC2016438H (R2)). Additionally, we obtained written informed consent from all participants before beginning the study.

### Anthropometric measurements

Trained researchers used standard procedures to collect anthropometric measurements. Height was measured precisely to 0.1 cm without shoes, utilizing the standard stadiometer. Weight was measured to the nearest 0.1 kg in participants wearing light indoor clothing. WC was determined as the midpoint between the rib margin and anterior superior iliac crest level and accurately measured to 0.1 cm. WC/height was used to calculate WHtR. BMI was determined by dividing the weight of the participant in kilograms by the square of their height in meters. ABSI, BRI, and CI were calculated using the formulas listed below [22, 28, 29].

ABSI = WC/(BMI^2/3^ ×height^1/2^).

BRI = 364.2-365.5 [1- π^−2^ WC^2^ (m) Height^− 2^ (m)]^1/2^.

CI = 0. 109^− 1^ WC (m) [Weight (kg)/Height (m)]^−1/2^.

### Definitions of outcomes

The primary outcome of the study was CMM, which was defined as the presence of at least two of the following medical conditions: CHD, stroke, hypertension, and diabetes [6]. Participants were categorized as having CHD or stroke if they had self-reported the condition during enrollment or had inpatient medical records indicating the diagnosis prior to enrollment. In a structured questionnaire administered at enrolment, the question “Have you ever been told by your doctor that you have CHD or stroke?“ was used to collect self-reported CHD and stroke data. Also, trained personnel identified participants’ CHD or stroke-related inpatient records from the Inpatients Registry before to recruitment using the I20–I25 codes for CHD and I61–I64 codes for stroke from the Tenth Revision of the International Classification of Diseases [30]. Hypertension was defined as systolic blood pressure (SBP) ≥ 140 mmHg, or/and diastolic blood pressure (DBP) ≥ 90 mmHg, or a self-reported history of hypertension, or use of antihypertensive drugs in the previous two weeks [31]. Participants rested for five minutes in a seated position before having their blood pressure checked twice on the right upper arm using an electronic blood pressure monitor (Omron HEM-7430; Omron Corporation, Kyoto, Japan). The two measurements of blood pressure were averaged to obtain a single value, which was then used in the analysis. In cases where a discrepancy exceeding 5 mmHg was found between the two blood pressure measurements, the procedure was repeated, and the mean value of three readings was taken as the final value [25]. Diabetes was defined as the use of hypoglycemic agents, self-reported history of diabetes, or having a fasting blood glucose level of ≥ 7.0 mmol/L [32].

### Covariates

A structured questionnaire was utilized, administered face-to-face, to gather data on various aspects. These Variables included sociodemographic details such as age, sex, occupation, residence, marriage, educational status, household income, and medical insurance, in addition to lifestyle behaviors (current smoking and current drinking) and medication usage. We also measured heart rate using standard protocols. Education status was categorized into “junior high school and below” and “high school and above”. Household income was divided into annual income greater than or equal to 50,000 yuan and annual income less than 50,000 yuan. The status of current smoking and current drinking among participants was determined based on self-reported data. In a personal interview, for instance, a participant was asked how frequently he/she had consumed alcohol in the previous year. If the participant responded, “never” the status was “No” but regardless of how frequently they had consumed alcohol, the status was “Yes”. The medication variable comprised the use of several types of drugs, including statins, lipid-lowering, antidiabetic, antiplatelet, and antihypertensive agents. Following the protocol of the China PEACE MPP project [25, 33], participants underwent lipid measurements including total cholesterol (TC), triglyceride (TG), low-density lipoprotein cholesterol (LDL-C), and high-density lipoprotein cholesterol (HDL-C), which were performed by a rapid lipid analyzer (CardioChek PA Analyzer; Polymer Technology Systems) using whole blood samples in their fasting state. Fasting blood glucose was measured using fingertip capillary blood samples.

### Statistical analysis

Based on the presence or absence of CMM, the analyzed population’s baseline characteristics were divided into two groups. Continuous variables were presented as medians (interquartile range) because their non-normal distribution was scrutinized using the Wilcoxon Rank-Sum Test. Number (percentage) was used to present categorical variables, and their comparison was conducted through appropriate methods such as the Wilcoxon Mann-Whitney test, Kruskal-Wallis H-test, or chi-square tests. We conducted multicollinearity test using tolerance and variance inflation factor to test for collinearity among covariates. According to the results of the multicollinearity test (**Table **S1), we created three groups of multivariate logistic regression models to compute the odds ratios (OR) with 95% confidence interval to investigate the associations of the six obesity-related anthropometric indices with the four single cardiometabolic diseases (hypertension, diabetes, CHD, and stroke) and CMM. Model 1 was unadjusted. Model 2 was solely adjusted for sex and age. Model 3 was further adjusted for sex, age, occupation, residence, marriage, educational status, household income, medical insurance, smoking, drinking, heart rate, medication use (lipid-lowering drugs, statins, and antiplatelet drugs), TC and HDL-C. Statistical differences between ORs were explored with a z-test [34]. Next, the correlation between the six adiposity indices and CMM was also examined using restricted cubic spline [35]. ROC (receiver operating characteristic) curve analysis was employed for each indicator to identify the maximum value of the sum of sensitivity and specificity, in order to determine its efficacy for identifying CMM. Discriminative power was calculated by using the area under the curve (AUC). Statistical differences between areas under the curve were tested with DeLong’s method, using BMI as the reference [34]. The point on the curve with the highest Youden index (sensitivity + specificity − 1) was the best cut-off point for each fat index when detecting CMM. Furthermore, we conducted subgroup analysis, including sex (men or women) and age (< 65 or ≥ 65 years). Finally, C-statistic and net reclassification index were employed to evaluate the incremental contribution of each adiposity index in discriminating CMM, in comparison to a base model. The basic model of CMM is comprised of several factors, including age, gender, occupation, place of residence, marriage, education level, household income, medical insurance, smoking, drinking, heart rate, TC, HDL-C, lipid-lowering medications, antiplatelet drugs, and statins. We conducted all analyses using R statistical software version 4.2.2 (R Project for Statistical Computing). A two side *P* value < 0.05 was considered to be statistically significant.

## Results

### Baseline characteristics

The study analyzed 101,973 participants, of which women constituted 60.5% and the median age was 54.0 years. Table 1 provided the baseline participant characteristics divided according to their CMM status. The overall crude prevalence of CMM was 11.5%. We detected significant differences between groups in all baseline characteristic variables, except for those related to medical insurance, residence, and current smoking (*P* < 0.05). Participants with CMM exhibited a higher likelihood of being farmers or alcohol drinkers, having higher levels of adiposity indices (BMI, WC, WHtR, BRI, and CI), fasting blood glucose, and TG, and taking medication (lipid-lowering drugs, statins, and antiplatelet drugs) compared to those without CMM. Additionally, participants with CMM had a higher prevalence of hypertension, CHD, stroke, and diabetes, and were generally older. Conversely, it was found that participants with CMM had a lower percentage of women, marriage, educational status, and household income compared to participants without CMM. In addition, participants with CMM had lower levels of TC, LDL-C, and HDL-C than those without CMM.

**Table 1:** Baseline characteristics among study participants with and without cardiometabolic multimorbidity

Characteristic	Overall	Non-CMM	CMM	*P* value
Number	101,973	90,215	11,758	
Age (years)	54.0 (46.0–63.0)	53.0 (45.0–62.0)	61.0 (54.0–67.0)	< 0.001
Women, n (%)	61,651 (60.5)	55,233 (61.2)	6418 (54.6)	< 0.001
Occupation (farmer), n (%)	11,990 (11.8)	10,379 (11.5)	1611 (13.7)	< 0.001
Residence (urban), n (%)	49,314 (48.4)	43,608 (42.8)	5706 (48.5)	0.697
Marriage (married), n (%)	92,273 (90.5)	81,831 (90.7)	10,442 (88.8)	< 0.001
Educational status (high school or above), n (%)	30,209 (29.6)	27,535 (30.5)	2674 (22.7)	< 0.001
Household income (50 000 yuan or above), n (%)	46,272 (45.4)	41,350 (45.8)	4922 (41.9)	< 0.001
Medical insurance, n (%)	95,100 (93.3)	84,090 (93.2)	11,010 (93.6)	0.085
Current smoking, n (%)	17,559 (17.2)	15,476 (17.2)	2083 (17.7)	0.133
Current drinking, n (%)	5415 (5.3)	4624 (5.1)	791 (6.7)	< 0.001
FBG, mg/dL	100.8 (90.0-113.4)	97.2 (88.2-109.8)	133.2 (115.2-156.6)	< 0.001
TC, mg/dL	186.0 (157.3-217.7)	186.0 (157.7-217.3)	184.8 (151.5–220.0)	< 0.001
TG, mg/dL	119.6 (87.7-171.9)	117.0 (85.9-166.6)	144.4 (102.8-211.8)	< 0.001
LDL-C, mg/dL	101.7 (78.1-128.4)	102.1 (78.9-128.4)	99.4 (72.3-129.1)	< 0.001
HDL-C, mg/dL	54.9 (44.5–67.3)	55.3 (44.8–67.7)	50.6 (42.1–61.5)	< 0.001
Heart rate, beats/min	76.5 (70.5–83.5)	76.0 (70.0–83.0)	78.5 (71.5–86.5)	< 0.001
BMI, kg/m^2^	23.9 (21.8–26.2)	23.7 (21.7–26.0)	25.4 (23.2–27.6)	< 0.001
WC, cm	83.0 (77.0–90.0)	83.0 (76.0–89.0)	89.0 (83.0–95.0)	< 0.001
WHtR	0.52 (0.49–0.56)	0.52 (0.48–0.56)	0.56 (0.52–0.59)	< 0.001
ABSI, m^**7/6**^/kg^**2/3**^	0.80 (0.76–0.83)	0.79 (0.76–0.83)	0.81 (0.78–0.84)	< 0.001
BRI	3.81 (3.09–4.61)	3.73 (3.03–4.51)	4.46 (3.73–5.31)	< 0.001
CI, m^**2/3**^/kg^2/3^	1.24 (1.18–1.29)	1.23 (1.18–1.29)	1.28 (1.23–1.33)	< 0.001
Current use of antihypertensive drugs, n (%)	19,822 (19.4)	12,667 (14.0)	7155 (60.9)	< 0.001
Current use of antidiabetic drugs, n (%)	7015 (6.9)	2433 (2.7)	4582 (39.0)	< 0.001
Current use of statins, n (%)	687 (0.7)	408 (0.5)	279 (2.4)	< 0.001
Current use of antiplatelet drugs, n (%)	473 (0.5)	234 (0.3)	239 (2.0)	< 0.001
Current use of lipid-lowering drugs, n (%)	3909 (3.8)	2314 (2.6)	1595 (13.6)	< 0.001
CHD, n (%)	2020 (2.0)	455 (0.5)	1565 (13.3)	< 0.001
Stroke, n (%)	2220 (2.2)	426 (0.5)	1794 (15.3)	< 0.001
Hypertension, n (%)	40,710 (39.9)	29,258 (32.4)	11,452 (97.4)	< 0.001
DM, n (%)	16,372 (16.1)	6461 (7.2)	9911 (84.3)	< 0.001

Data was presented as median (inter quartile range) for non-normally distributed variables, and number (percentage) for categorical variables

Abbreviations: CMM, cardiometabolic multimorbidity; BMI, body mass index; WC, waist circumference; WHtR, waist-to-height ratio; ABSI, a body shape index; BRI, body roundness index; CI, conicity index; FBG, fasting blood glucose; TC, total cholesterol; LDL-C, low density lipoprotein-cholesterol; HDL-C, high density lipoprotein-cholesterol; TG, triglyceride; CHD: coronary heart disease; DM, diabetes

Cite and indicate: Table 1 should placed at the end of the baseline Characteristics section on page 14 of the text file

### Association of adiposity indices and CMM

**Fig. 1:**
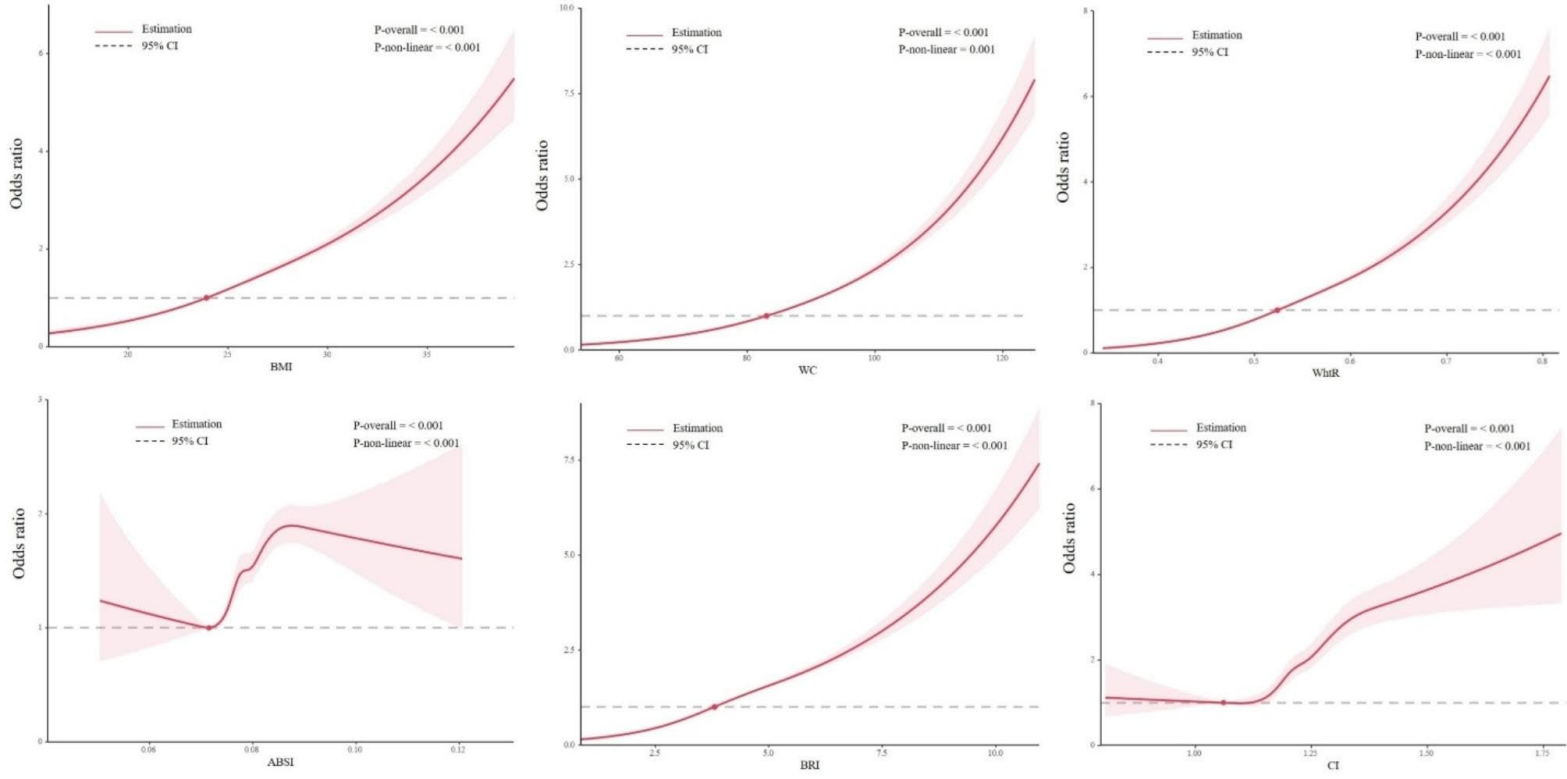
**The associations between adiposity indices and cardiometabolic multimorbidity by restricted cubic spline** Abbreviations: BMI, body mass index; WC, waist circumference; WHtR, waist-to-height ratio; ABSI, a body shape index; BRI, body roundness index; CI, conicity index

**Table S2** showed the fully-adjusted ORs (95% CIs) for the four single cardiometabolic diseases of the six anthropometric indices. We found that all six indices were significantly associated with every single cardiometabolic disease including hypertension, diabetes, CHD, and stroke. The associations between adiposity indices and CMM were presented in Table 2. Adjusting for sex, age, occupation, residence, marriage, educational status, household income, medical insurance, smoking, drinking, heart rate, TC, HDL-C, lipid-lowering drugs, antiplatelet drugs, and statins, we found significant associations between all adiposity indices and CMM. Our findings showed larger ORs for CMM with WC, WHtR, and BRI compared with BMI. The z-test indicated that there were significant differences among the OR values (*P* < 0.05). **Table S3** and **Table S4** compared the ORs for six adiposity indices stratified by sex and age groups, respectively. Men had higher ORs than women at each adiposity indices. WC showed highest OR 1.61 (1.56–1.66) for women, while the WHtR had highest OR 1.72 (1.66–1.79) for men in model 3 (z-test *P* < 0.05) (**Table S3**). For each measure, middle-aged individuals had higher ORs associated with CMM than older individuals, with WC having higher ORs in both middle-aged people and elderly people in multiple logistic regression models than BMI (z-test *P* < 0.05) (**Table S4**). Figure 1 presented evidence of a non-linear association between the odds of CMM and elevated adiposity indices, with higher values in all indices being linked to an increased probability of CMM.

**Table 2:** The associations between adiposity indices and cardiometabolic multimorbidity by multivariate logistic regression analysis

	BMI	WC	WHtR	ABSI	BRI	CI
Model 1	1.58 (1.55–1.62)	1.87 (1.83–1.91)	1.87 (1.84–1.91)	1.48 (1.45–1.51)	1.80 (1.77–1.84)	1.74 (1.71–1.78)
Model 2	1.64 (1.61–1.68)	1.77 (1.73–1.81)	1.72 (1.68–1.76)	1.22 (1.19–1.25)	1.67 (1.64–1.70)	1.47 (1.43–1.50)
Model 3	1.55 (1.52–1.58)	1.66 (1.62–1.70) *	1.61 (1.58–1.65) *	1.17 (1.15–1.20) *	1.57 (1.53–1.60)	1.38 (1.35–1.41) *

Model 1 adjust for none

Model 2 adjust for age and sex

Model 3 adjust for age, sex, occupation, residence, marriage, educational status, household income, medical insurance, smoking, drinking, heart rate, total cholesterol, high-density lipoprotein cholesterol, antiplatelet drugs, statins, and lipid-lowering drugs

Abbreviations: BMI, body mass index; WC, waist circumference; WHtR, waist-to-height ratio; ABSI, a body shape index; BRI, body roundness index; CI, conicity index

* *P* < 0.05, which is considered a statistical difference between ORs tested with z-test using BMI as the reference

Cite and indicate: Table 2 should placed at the end of the association of adiposity indices and CMM section on page 15 of the text file

### AUCs for CMM in different adiposity indices

The AUCs (95% CIs) and optimal cut points for adiposity indices in relation to CMM were shown in Table 3. When compared to BMI, WHtR and BRI tended to have higher and equally discriminative value for CMM (AUCs: 0.679, sensitivity 64.1%, specificity 62.3%, cutoff point: 0.537 vs. AUCs: 0.679, sensitivity 64.1%, specificity 62.3%, cutoff point: 4.077, respectively). The discriminative power for each adiposity parameter stratified by sex and age were presented in **Table S5** and **Table S6**, respectively. For men and women, WHtR and BRI had greater AUCs for detecting CMM compared with BMI (**Table S5**). WC had the higher discriminative power for middle-aged people (AUC: 0.695, sensitivity 68%, specificity 60.5%, cutoff point: 84.95 cm) and elderly population (AUC: 0.638, sensitivity 59.6%, specificity 60.5%, cutoff point: 86.55 cm) for CMM compared with BMI (**Table S6**). The Delong’s method indicated that there were significant differences among the AUC values (*P* < 0.05).

**Table 3:** AUCs and optimal cut points for adiposity indices in relation to cardiometabolic multimorbidity

	AUC (95% CI)	Cut-off point	Sensitivity	Specificity	Youden index	*P*-value*
BMI	0.637 (0.632–0.643)	24.437	0.616	0.587	0.203	/
WC	0.675 (0.670–0.680)	85.75	0.636	0.620	0.256	0.023
WHtR	0.679 (0.675–0.684)	0.537	0.641	0.623	0.264	0.019
ABSI	0.620 (0.614–0.625)	0.080	0.589	0.587	0.176	0.041
BRI	0.679 (0.675–0.684)	4.077	0.641	0.623	0.264	0.018
CI	0.660 (0.655–0.665)	1.251	0.651	0.585	0.236	0.048

Abbreviations: AUC, area under the receiver operating characteristic curve; CI, confidence interval; BMI, body mass index; WC, waist circumference; WHtR, waist-to-height ratio; ABSI, a body shape index; BRI, body roundness index; CI, conicity index

* P-value < 0.05 is considered a statistical difference between AUCs tested with DeLong’s method (compare with BMI).

Cite and indicate: Table 3 should placed at the end of the AUCs for CMM in different adiposity indices section on page 16 of the text file

### Incremental value of adiposity indices for the discrimination of CMM

Incremental effect of adiposity indices surrogates on CMM was showed in Table 4. When compared with BMI, adding WC, WHtR, or BRI to a base model provided larger incremental values for the discrimination of CMM (all *P* < 0.05). These were demonstrated by the increase in the C-statistic, with the base model having a C-statistic of 0.750 (95% CI: 0.746–0.754), and the models including WC, WHtR, and BRI having C-statistics of 0.777 (95% CI: 0.773–0.781), 0.775 (95% CI: 0.771–0.779), and 0.775 (95% CI: 0.768–0.776), respectively. Additionally, the net reclassification index values were 0.374 (0.365–0.381) for BMI, 0.388 (95% CI: 0.380–0.420) for WC, 0.380 (95% CI: 0.369-0.400) for WhtR, and 0.370 (95% CI: 0.352–0.393) for BRI.

**Table 4:** Incremental value of adiposity indices for the discrimination of cardiometabolic multimorbidity

	C-statistic (95% confidence interval)	*P* value	Net reclassification index (95% confidence interval)	*P* value
Base model *	0.750 (0.746–0.754)	/	/	/
+BMI	0.773 (0.769–0.777)	< 0.001	0.374 (0.365–0.381)	< 0.001
+WC	0.777 (0.773–0.781)	< 0.001	0.388 (0.380–0.420)	< 0.001
+WHtR	0.775 (0.771–0.779)	< 0.001	0.380 (0.369-0.400)	< 0.001
+ABSI	0.753 (0.749–0.756)	0.046	0.138 (0.119–0.142)	< 0.001
+BRI	0.775 (0.768–0.776)	< 0.001	0.370 (0.352–0.393)	< 0.001
+CI	0.762 (0.758–0.766)	< 0.001	0.259 (0.251–0.287)	< 0.001

* Base model: age, sex, occupation, residence, marriage, educational status, household income, medical insurance, smoking, drinking, heart rate, total cholesterol, high-density lipoprotein cholesterol, antiplatelet drugs, statins, and lipid-lowering drugs

Abbreviations: BMI, body mass index; WC, waist circumference; WHtR, waist-to-height ratio; ABSI, a body shape index; BRI, body roundness index; CI, conicity index

Cite and indicate: Table 4 should placed at the end of the incremental value of adiposity indices for the discrimination of CMM section on page 16 of the text file

## Discussion

The present study was conducted on a large sample of Chinese adults aged 35 to 75 years to examine the relationship between six adiposity indices (BMI, WC, WHtR, ABSI, BRI, and CI) and CMM using a cross-sectional design. our study revealed that six obesity indices were all significantly and independently associated with the odds of CMM in the Chinese population, but the strength of these associations varied. In particular, when compared with BMI, WC and WHtR showed stronger associations with CMM, and WC, WHtR, and BRI were better discriminative power for CMM.

CMM is a significant issue that is challenging healthcare systems all around the world [7–9]. The prevalence of CMM in this study was 11.5%, consistent with the study by Sewpaul R et al. [8]. Previous researches have explored that CMM is more harmful than the absence or presence of cardiometabolic disease alone [2, 7]. Despite the increasing burden of CMM and staggering health impairments, existing studies have mainly focused on individual cardiometabolic disease, and the number of studies investigating the relationship between easily accessible obesity indices (such as BMI, WC, WHtR, ABSI, BRI, and CI) and CMM is very limited. To our knowledge, few studies have examined the relationship between BMI and CMM [11, 18, 36]. A comprehensive analysis revealed that rising BMI was associated with an elevated risk of cardiometabolic comorbidity; compared with people with a healthy weight, the risk of developing CMM in overweight people was twice as high, almost five times higher for people with moderately obesity, and almost fifteen times higher for people in very obese [11]. Another study involving 8,270 European population showed that overweight/obesity was positively associated with the single cardiometabolic diseases and CMM [36]. In addition, Mika Kivimäki et al. Classification of obesity into stages 1, 2, and 3 was found to show a dose-response pattern; the higher the degree of obesity, the greater the relative risk of comorbidities, which was consistent to our study [13]. However, these studies did not include Asian populations, and did not analyze the relationship between other obesity indicators and comorbidities except BMI, and our research has produced some new findings for this. According to a study by Lu Yet et al., WC, WHtR, and BMI were all independent predictors of CMM in Chinese people, with WC and WHtR being more so than BMI [18], which was similar to our findings. However, due to limited sample size, missing subgroup analysis, and lack of adjustment for potential CMM influencing factors such as marriage and economic income [36, 37], the results of this study may not be definitive. Therefore, the relationship between WC, WHtR, and CMM needs to be further confirmed in a representative large sample study. Our study added to the existing body of knowledge by focusing on the Chinese adults and examining the association between WC, WHtR, BRI, CI, and ABSI with CMM in comparison with BMI.

Notably, in our study, WHtR and BRI were more strongly associated with CMM than BMI, and superior to BMI in discriminative ability for CMM among Chinese adults, which was consistent to the previous research [38]. We also found that the optimal BRI thresholds for detecting CMM were 3.8 in men and 4.1 in women, which is in good agreement with a previous Chinese study that measured a BRI of 3.9 in overweight men and 4.0 in women [24]. Nkwana MR et al. pointed out that CI was positively associated with metabolic disorder and risk factors for cardiovascular disease [39]. While the research of Haoyu Wang et al. suggested that CI was not as potent as BMI in identifying metabolic syndrome and its components in Chinese adults [22]. However, their study was based on a small sample size, which may affect the robustness of the results. Alemedia et al. recommended a cut-off point of 1.25 for CI as indicators of increased occurrence of cardiovascular risk factors [19], the similar result was also observed in our study of overall. In northeast China, study by Chang Y et al. showed that ABSI was not superior to BMI in discriminative ability for cardiovascular risk factors [21], which was consistent to our study.

This study showed that WC, WHtR, and BRI were better discriminative performance for the risk of CMM than BMI. This phenomenon can be attributed to several factors, the primary one being that BMI is limited in its ability to differentiate between the location of peripheral versus central fat [15]. The risk of developing cardiometabolic diseases can vary among individuals with similar BMI [40]. This may be because susceptibility to cardiometabolic diseases associated with obesity is not solely caused by general body fat, but rather influenced by variations in regional fat distribution and the impact of subcutaneous adipose tissue [40]. In addition, compared with BMI, WC is more accurate in reflecting body fat percentage and may play a critical part in predicting early onset metabolic abnormalities [41, 42]. Ashwell M et al. pointed out that WC improved the power to detect adverse cardiometabolic risk outcomes by 3% compared with BMI [43]. Furthermore, research has shown that waist-to-height ratio (WHtR) is a relatively stable and gender-independent indicator of cardiometabolic risk, as it is not significantly influenced by factors such as age or race [44, 45]. In fact, individuals with a normal BMI but a high WHtR may be at increased risk of developing cardiometabolic diseases [40]. This is supported by recent findings which suggested that WHtR may be more effective than BMI in distinguishing adverse cardiometabolic risk effects, with a potential improvement of 4–5% [43]. The use of BRI as a predictor could enhance the accuracy of predicting body fat percentage and visceral adiposity tissue when compared to traditional metrics such as BMI [29]. The findings of several studies indicated that BRI has greater predictive power than BMI for risks associated with individual cardiometabolic and cardiovascular diseases [21, 46].

The exact mechanism between the obesity indicators and CMM remains to be elucidated, and the ectopic fat pool may be one of the important reasons [47]. First, as obesity progresses, ectopic fat is infiltrated by macrophages and some adipokines are upregulated [48]. Research has demonstrated the importance of these adipokines in the pathogenesis of inflammation and insulin resistance (IR) [48]. Second, the accumulation of adipose tissue in organs involved in glucose, insulin, and lipid metabolism is considered a direct cause of metabolic disturbance. As the adipose tissue accumulates in these critical organs, it can have systemic effects and lead to a range of metabolic diseases that affect the entire body. The presence of fat in these organs can disrupt normal metabolic function, and ultimately lead to the development of systemic metabolic disorders such as type 2 diabetes and cardiovascular disease [14, 49]. Body mass index (BMI) does not reflect the differentiation of ectopic fat nor fully capture the complex biology of obesity [14], which may result in BMI being not the best discriminator of cardiometabolic comorbidities. Moreover, IR may be a central mechanism of cardiometabolic disorders [50], and IR may inhibit crucial metabolic pathways and stimulate growth factors, potentially leading to cardiometabolic disease and impairing normal cardiac function [51, 52].

The study’s large population offered significant strength, allowing for more precise analyses and increasing the study’s generalizability. Further, our study was the first to compare obesity-related anthropometric parameters (such as BMI, WC, WHtR, BRI, ABSI, and CI) in discriminative ability for CMM in Chinese adults aged 35–75. Limitations should also be noted. First, the current study’s cross-sectional design restricts its capacity to establish causality, and therefore, future research using prospective cohort studies will be necessary to confirm any causative relationship suggested by the research. Second, misdiagnosis of diabetes might exist due to the blood glucose measurements using fingertip samples rather than serum blood samples. Thus, caution should be noted when generalizing our conclusions because of diagnosis bias. Further studies that use whole blood glucose to diagnose diabetes and subsequent CMM are needed to confirm our results. Third, it should be acknowledged that the study’s findings are based on research conducted solely on Chinese adults and, therefore, may not be generalizable to other cultural or ethnic populations. Finally, there were some uncollected CMM-related indicators (such as physical exercise and dietary patterns) that were not adjusted in the model, and these indicators may lead to confounding and thus affect our results.

## Conclusions

To summarize, our study found that obesity-related anthropometric measures were positively associated with CMM, with WC and WHtR demonstrating stronger association than BMI. Moreover, WC, WHtR, and BRI were superior to BMI in discriminative ability for CMM in Chinese subjects. The evidence highlights the importance of preventative measures focused on managing obesity, particularly abdominal obesity, as the primary preventative strategy for CMM while controlling other major CMM risk factors.

### Electronic supplementary material

Below is the link to the electronic supplementary material.

Supplementary Material 1

## Data Availability

All data generated or analyzed during this study are included in this published article and its supplementary information files.

## Declarations

### Author information

QXR, Department of Cardiology, Zhuhai hospital affiliated with Jinan University (Zhuhai People’s Hospital), Zhuhai, China; Department of cardiology, Guangdong Cardiovascular Institute, Guangdong Provincial People’s Hospital (Guangdong Academy of Medical Sciences), Southern Medical University, Guangzhou, China, Email: qinxiaoru@gdph.org.cn; CCL, Department of cardiology, Guangdong Cardiovascular Institute, Guangdong Provincial People’s Hospital (Guangdong Academy of Medical Sciences), Southern Medical University, Guangzhou, China, Email: chenchaolei@gdph.org.cn; WJB, Global Health Research Center, Guangdong Provincial People’s Hospital (Guangdong Academy of Medical Sciences), Southern Medical University, Guangzhou, China, Email: wangjiabin@gdph.org.cn; CAP, Department of cardiology, Guangdong Cardiovascular Institute, Guangdong Provincial People’s Hospital (Guangdong Academy of Medical Sciences), Southern Medical University, Guangzhou, China, Email: acai0120@hotmail.com; FXX, Guangdong Provincial People’s Hospital (Guangdong Academy of Medical Sciences), Southern Medical University, Email: fengxiaoxuan@gdph.org.cn; JXF, Department of Cardiology, Zhuhai People’s Hospital (Zhuhai hospital affiliated with Jinan University), Zhuhai, China, Email: jxf_1982@sina.com.

## Abbreviations

CMM: cardiometabolic multimorbidity
BMI: body mass index
WC: waist circumference
WHtR: waist-to-height ratio
ABSI: a body shape index
BRI: body roundness index
CI: conicity index
FBG: fasting blood glucose
TC: total cholesterol
LDL-C: low density lipoprotein-cholesterol
HDL-C: high density lipoprotein-cholesterol
TG: triglyceride
CHD: coronary heart disease
DM: diabetes
ROC: receiver operating characteristic
AUC: area under the curve
OR: the odds ratios
CI: confidence interval
