# Supplementary material for: Association of adiposity indices with cardiometabolic multimorbidity among 101,973 chinese adults: a cross-sectional study
Source: BMC Cardiovasc Disord. 2023 Oct 21;23:514. doi: 10.1186/s12872-023-03543-x (PMC10590510; doi:10.1186/s12872-023-03543-x)
Supplement: Supplementary file 1 — Supplementary Material 1 [file 12872_2023_3543_MOESM1_ESM.doc]

**SUPPLEMENTAL MATERIAL**

**Table S1** Multicollinearity test among covariates

| **Covariates** | **Collinearity Statistics *** | |
| --- | --- | --- |
|  | **Tolerance** | **VIF** |
| Age | 0.861 | 1.162 |
| Women | 0.632 | 1.582 |
| Occupation | 0.923 | 1.083 |
| Residence | 0.948 | 1.055 |
| Marriage | 0.984 | 1.016 |
| Educational status | 0.858 | 1.165 |
| Household income | 0.940 | 1.063 |
| Medical insurance | 0.968 | 1.033 |
| Current smoking | 0.687 | 1.455 |
| Current drinking | 0.919 | 1.088 |
| Total cholesterol | 0.467 | 2.142 |
| Triglyceride | 0.075 | 13.272 |
| Low-density lipoprotein cholesterol | 0.096 | 10.417 |
| High-density lipoprotein cholesterol | 0.376 | 2.660 |
| Heart rate | 0.977 | 1.024 |
| Current use of statins | 0.758 | 1.320 |
| Current use of antiplatelet drugs | 0.887 | 1.128 |
| Current use of lipid-lowering drugs | 0.818 | 1.222 |

***** Multicollinearity exists when tolerance < 0.1 and/or VIF > 10. VIF indicates variance inflation factor.

**Table S2** Multivariate analysis of the associations of adiposity indices with single cardiometabolic disease

|  | **Hypertension** | **Diabetes** | **CHD** | **Stroke** |
| --- | --- | --- | --- | --- |
|  | **OR (95% CI)** | **OR (95% CI)** | **OR (95% CI)** | **OR (95% CI)** |
| **BMI** | 1.64 (1.62-1.67) | 1.39 (1.37-1.42) | 1.12 (1.07-1.17) | 1.07 (1.02-1.12) |
| **WC** | 1.63 (1.61-1.66) | 1.50 (1.48-1.53) | 1.16 (1.11-1.22) | 1.13 (1.08-1.18) |
| **WHtR** | 1.60 (1.58-1.63) | 1.47 (1.44-1.50) | 1.15 (1.10-1.21) | 1.13 (1.08-1.18) |
| **ABSI** | 1.07 (1.06-1.09) | 1.18 (1.15-1.20) | 1.08 (1.03-1.14) | 1.11 (1.06-1.16) |
| **BRI** | 1.59 (1.56-1.61) | 1.44 (1.42-1.47) | 1.14 (1.09-1.19) | 1.12 (1.08-1.17) |
| **CI** | 1.27 (1.25-1.29) | 1.33 (1.30-1.36) | 1.12 (1.07-1.18) | 1.14 (1.09-1.19) |

Models were adjusted for age, sex, occupation, residence, marriage, educational status, household income, medical insurance, smoking, drinking, heart rate, total cholesterol, high-density lipoprotein cholesterol, antiplatelet drugs, statins, and lipid-lowering drugs.

OR, odds ratio; CI, confidence interval; BMI, body mass index; WC, waist circumference; WHtR, waist-to-height ratio; ABSI, a body shape index; BRI, body roundness index; CI, conicity index.

**Table S3** The associations between adiposity indices and CMM by multivariate logistic regression analysis, stratified by sex

|  | **BMI** | **WC** | **WHtR** | **ABSI** | **BRI** | **CI** |
| --- | --- | --- | --- | --- | --- | --- |
| **Men** |  |  |  |  |  |  |
| Model 1 | 1.54 (1.49-1.58) | 1.75 (1.70-1.81) | 1.88 (1.82-1.94) | 1.45 (1.40-1.49) | 1.83 (1.78-1.89) | 1.73 (1.67-1.79) |
| Model 2 | 1.70 (1.65-1.76) | 1.82 (1.76-1.88) | 1.84 (1.78-1.91) | 1.29 (1.25-1.34) | 1.80 (1.75-1.86) | 1.58 (1.53-1.64) |
| Model 3 | 1.60 (1.55-1.66) | 1.71 (1.65-1.77) * | 1.72 (1.66-1.79) * | 1.23 (1.19-1.28) * | 1.69 (1.63-1.75) * | 1.48 (1.42-1.53) * |
| **Women** | | | | | | |
| Model 1 | 1.62 (1.58-1.66) | 1.98 (1.93-2.04) | 1.94 (1.89-1.99) | 1.48 (1.45-1.52) | 1.85 (1.81-1.89) | 1.74 (1.69-1.78) |
| Model 2 | 1.59 (1.55-1.63) | 1.71 (1.66-1.76) | 1.63 (1.58-1.67) | 1.15 (1.12-1.18) | 1.57 (1.53-1.61) | 1.37 (1.33-1.41) |
| Model 3 | 1.51 (1.47-1.55) | 1.61 (1.56-1.66) * | 1.53 (1.49-1.57) | 1.11 (1.08-1.14) * | 1.48 (1.44-1.52) | 1.30 (1.26-1.33) * |

Model 1 adjust for none.

Model 2 adjust for age.

Model 3 adjust for age, occupation, residence, marriage, educational status, household income, medical insurance, smoking, drinking, heart rate, total cholesterol, high-density lipoprotein cholesterol, antiplatelet drugs, statins, and lipid-lowering drugs.

Abbreviations: BMI, body mass index; WC, waist circumference; WHtR, waist-to-height ratio; ABSI, a body shape index; BRI, body roundness index; CI, conicity index; CMM, cardiometabolic multimorbidity.

* *P* < 0.05 is considered a statistical difference between ORs tested with z-test using BMI as the reference.

**Table S4** The associations between adiposity indices and CMM by multivariate logistic regression analysis, stratified by age

|  | **BMI** | **WC** | **WHtR** | **ABSI** | **BRI** | **CI** |
| --- | --- | --- | --- | --- | --- | --- |
| **Age < 60 years old** |  |  |  |  |  |  |
| Model 1 | 1.74 (1.69-1.79) | 1.99 (1.94-2.05) | 1.98 (1.92-2.04) | 1.42 (1.38-1.46) | 1.90 (1.85-1.96) | 1.75 (1.70-1.80) |
| Model 2 | 1.72 (1.67-1.77) | 1.87 (1.81-1.93) | 1.84 (1.79-1.90) | 1.24 (1.20-1.28) | 1.78 (1.73-1.84) | 1.53 (1.48-1.58) |
| Model 3 | 1.60 (1.55-1.65) | 1.73 (1.67-1.79) * | 1.70 (1.65-1.76) * | 1.18 (1.14-1.22) * | 1.65 (1.60-1.70) * | 1.42 (1.37-1.47) * |
| **Age ≥ 60 years old** | | | | | | |
| Model 1 | 1.54 (1.50-1.58) | 1.66 (1.61-1.71) | 1.58 (1.54-1.62) | 1.22 (1.19-1.25) | 1.53 (1.50-1.57) | 1.43 (1.39-1.47) |
| Model 2 | 1.56 (1.52-1.60) | 1.66 (1.61-1.71) | 1.60 (1.56-1.64) | 1.18 (1.15-1.22) | 1.55 (1.51-1.59) | 1.39 (1.35-1.43) |
| Model 3 | 1.48 (1.44-1.52) | 1.57 (1.52-1.62) * | 1.51 (1.47-1.56) | 1.15 (1.12-1.18) * | 1.47 (1.43-1.51) | 1.32 (1.29-1.36) * |

Model 1 adjust for none.

Model 2 adjust for age and sex.

Model 3 adjust for age, sex, occupation, residence, marriage, educational status, household income, medical insurance, smoking, drinking, heart rate, total cholesterol, high-density lipoprotein cholesterol, antiplatelet drugs, statins, and lipid-lowering drugs.

Abbreviations: BMI, body mass index; WC, waist circumference; WHtR, waist-to-height ratio; ABSI, a body shape index; BRI, body roundness index; CI, conicity index; CMM, cardiometabolic multimorbidity.

* *P* < 0.05, which is considered a statistical difference between ORs tested with z-test using BMI as the reference.

**Table S5** AUCs and optimal cut points for adiposity indices in relation to CMM, stratified by sex

|  | **Men (n=40322)** | | | | | **Women (n = 61651)** | | | | | |
| --- | --- | --- | --- | --- | --- | --- | --- | --- | --- | --- | --- |
|  | **AUC (95% CI)** | **Cutoff point** | **Sensitivity** | **Specificity** | **Youden index** | | **AUC (95% CI)** | **Cutoff point** | **Sensitivity** | **Specificity** | **Youden index** |
| **BMI** | 0.621 (0.613-0.629) | 24.448 | 0.632 | 0.545 | 0.177 | | 0.645 (0.638-0.652) | 24.568 | 0.585 | 0.632 | 0.217 |
| **WC** | 0.650 (0.643-0.658) * | 87.250 | 0.662 | 0.560 | 0.222 | | 0.687 (0.680-0.694) * | 82.850 | 0.683 | 0.590 | 0.273 |
| **WHtR** | 0.661 (0.653-0.668) * | 0.526 | 0.675 | 0.562 | 0.237 | | 0.698 (0.692-0.705) * | 0.540 | 0.663 | 0.632 | 0.295 |
| **ABSI** | 0.601 (0.593-0.609) * | 0.081 | 0.595 | 0.560 | 0.155 | | 0.629 (0.622-0.636) * | 0.079 | 0.646 | 0.546 | 0.192 |
| **BRI** | 0.661 (0.653-0.668) * | 3.865 | 0.675 | 0.562 | 0.237 | | 0.698 (0.692-0.705) * | 4.144 | 0.663 | 0.632 | 0.295 |
| **CI** | 0.640 (0.632-0.647) * | 1.269 | 0.588 | 0.619 | 0.207 | | 0.670 (0.663-0.677) * | 1.237 | 0.690 | 0.563 | 0.253 |

Abbreviations: AUC, area under the receiver operating characteristic curve; CI, confidence interval; BMI, body mass index; WC, waist circumference; WHtR, waist-to-height ratio; ABSI, a body shape index; BRI, body roundness index; CI, conicity index; CMM, cardiometabolic multimorbidity.

* *P*-value < 0.05, which is considered a statistical difference between AUCs tested with DeLong’s method (compare with BMI).

**Table S6** AUCs and optimal cut points for adiposity indices in relation to CMM, stratified by age

|  | **Age < 60 years old** **(n = 66172)** | | | | | **Age ≥ 60 years old (n = 35261)** | | | | | |
| --- | --- | --- | --- | --- | --- | --- | --- | --- | --- | --- | --- |
|  | **AUC (95% CI)** | **Cutoff point** | **Sensitivity** | **Specificity** | **Youden index** | | **AUC (95% CI)** | **Cutoff point** | **Sensitivity** | **Specificity** | **Youden index** |
| **BMI** | 0.667 (0.66-0.675) | 24.567 | 0.652 | 0.596 | 0.248 | | 0.625 (0.618-0.632) | 24.423 | 0.581 | 0.603 | 0.184 |
| **WC** | 0.695 (0.687-0.702) * | 84.950 | 0.680 | 0.605 | 0.285 | | 0.638 (0.630-0.645) * | 86.550 | 0.596 | 0.605 | 0.201 |
| **WHtR** | 0.690 (0.683-0.698) * | 0.518 | 0.747 | 0.529 | 0.276 | | 0.632 (0.625-0.640) * | 0.538 | 0.657 | 0.539 | 0.196 |
| **ABSI** | 0.605 (0.598-0.613) * | 0.078 | 0.671 | 0.488 | 0.159 | | 0.561 (0.554-0.568) * | 0.081 | 0.654 | 0.446 | 0.100 |
| **BRI** | 0.690 (0.683-0.698) * | 3.695 | 0.747 | 0.529 | 0.276 | | 0.632 (0.625-0.640) * | 4.099 | 0.657 | 0.539 | 0.196 |
| **CI** | 0.657 (0.650-0.665) * | 1.222 | 0.725 | 0.504 | 0.229 | | 0.601 (0.594-0.609) * | 1.254 | 0.695 | 0.456 | 0.151 |

Abbreviations: AUC, area under the receiver operating characteristic curve; CI, confidence interval; BMI, body mass index; WC, waist circumference; WHtR, waist-to-height ratio; ABSI, a body shape index; BRI, body roundness index; CI, conicity index; CMM, cardiometabolic multimorbidity.

* *P*-value < 0.05, which is considered a statistical difference between AUCs tested with DeLong’s method (compare with BMI).
